# Peri-Implant Marginal Bone Changes around Dental Implants with Platform-Switched and Platform-Matched Abutments: A Retrospective 5-Year Radiographic Evaluation

**DOI:** 10.3390/jpm12081226

**Published:** 2022-07-27

**Authors:** Hsi-Kuei Lin, Jerry Chin-Yi Lin, Yu-Hwa Pan, Eisner Salamanca, Yi-Ting Chang, Yung-Szu Hsu, Yi-Fan Wu, Chin-Kai Lin, Odontuya Dorj, Wei-Jen Chang

**Affiliations:** 1 School of Dentistry, College of Oral Medicine, Taipei Medical University, Taipei 110, Taiwan; linhsikuei38@gmail.com (H.-K.L.); drjerrylin@gmail.com (J.C.-Y.L.); shalom.dc@msa.hinet.net (Y.-H.P.); eisnergab@tmu.edu.tw (E.S.); nm8346@yahoo.com.tw (Y.-S.H.); yfwu@tmu.edu.tw (Y.-F.W.); 01728@km.eck.org.tw (C.-K.L.); 2 School of Oral Hygiene, College of Oral Medicine, Taipei Medical University, Taipei 110, Taiwan; 3 Dental Department, Taipei Medical University, Shuang-Ho Hospital, New Taipei City 235, Taiwan; 4 Department of Oral Medicine, Infection and Immunity, Harvard School of Dental Medicine, Boston, MA 01238, USA; 5 Department of Dentistry, Chang Gung Memorial Hospital, Taipei 105, Taiwan; 6 Graduate Institute of Dental and Craniofacial Science, Chang Gung University, Taoyuan 333, Taiwan; 7 School of Dentistry, College of Medicine, China Medical University, Taichung 404, Taiwan; 8 Department of Dentistry, En Chu Kong Hospital, New Taipei City 237, Taiwan; thesmurfylove@gmail.com; 9 Department of Dental Technology and Dental Hygiene, School of Dentistry, Mongolian National University of Medical Sciences, Ulaanbaatar 14210, Mongolia

**Keywords:** dental implants, dental prostheses, marginal bone loss, natural teeth, opposing structure, platform-switched, platform-matched

## Abstract

Preserving the marginal bone level (MBL) is essential for the long-term success of dental implant therapy, and bone remodeling around dental implants is considered to vary with time. Numerous studies comparing the platform-switching (PS) and platform-matching (PM) dental implants have indicated that PS dental implants showed a lesser reduction for the MBL, and the majority of them had a relatively short period. This study aimed to evaluate vertical and horizontal bone defects by using digital periapical radiographs to examine the changes in MBL around PM and PS dental implants over 5 years after functional loading. The vertical MBL (vMBL) was measured from the implant–abutment junction to the first bone-to-implant contact. The horizontal MBL (hMBL) was measured from the implant–abutment junction to the bone crest. All data were presented as means ± standard errors. Paired and independent *t*-tests with Welch’s correction were used to analyze the data. A total of 61 dental implants in 38 patients after 5 years of functional loading were evaluated. Over time, PS dental implants were more likely to gain bone; by contrast, PM dental implants were more likely to lose bone during the observation time. Changes in vMBL for PS dental implants were significantly less than those for PM dental implants at 1-year (*p* = 0.045), 3-year (*p* = 0.021), and 5-year (*p* = 0.010) loading. Likewise, changes in hMBL for PS dental implants were significantly smaller than in those for PM dental implants at 3-year (*p* = 0.021) and 5-year (*p* = 0.006) loading; however, the changes were minimal in both approaches. PS dental implants had a significant increment in the percentage of bone integration, whereas that for PM dental implants dropped over time, with no significance. In PS dental implants that occlude with natural teeth, vertical and horizontal bone gain was observed, and it was significant at 3 years (*p* = 0.023). A significant horizontal bone gain was observed in the opposing natural teeth at 3-year (*p* = 0.002) and 5-year loading (*p* = 0.002). The PS concept appears to preserve more MBL around dental implants by stabilizing the vMBL and hMBL over a 5-year period. A minimal marginal bone change was detected in both concepts. The opposing natural teeth at PS dental implants showed a favorable effect on marginal bone tissues.

## 1. Introduction

From the literature, the platform-switching (PS) method is a crucial factor that lessens the marginal bone level (MBL), and the biological width contributes to the maintenance of hard and soft tissues. The implant–abutment junction is positioned internally in the PS approach. For the preservation of the marginal bone, internal positioning makes inflammatory invasion distant from the bone and forms a plane of biological width. Moreover, this lessens bone-level reduction by altering the micro-space from the marginal bone [1]. Bone remodeling appeared to depend on a particular infectious area of the soft tissue surrounding the dental implant [2]. Another study reported that the implant–abutment junction was affected by infiltrating inflammation cells [3].

The existence of an adequate amount of bone and its quality can project the long-term success of dental implants. Crestal bone loss around dental implants is quite a common complication following an abutment connection. Adell et al. assessed the peri-implant bone level around dental implants in the first year after loading, with average bone loss of 1.2 mm [4], and found that the majority of peri-implant bone remodeling occurred during the first year of post-loading [5]. Another study evaluated the peri-implant bone surrounding the submerged dental implant and reported 1.5–2.0 mm of marginal bone loss after the first year of prosthetic installment [6].

Sesma et al. evaluated the 1-year post-functional loading of crestal bone loss around PS and platform-matched (PM) dental implants. Authors found a significant increment of horizontal and vertical bone losses around PS and non-PS dental implants (horizontal bone loss, 0.84 mm vs. 1.04 mm, *p* < 0.05; vertical bone loss, 0.82 mm vs. 0.99 mm, *p* < 0.05) [7]. A meta-analysis of 28 studies reported lesser MBL around PS dental implants than non-PS dental implants [8]. A systematic review and meta-analysis of 1239 dental implants showed that PS appears to preserve horizontal and vertical bone loss and soft tissue [9]. A randomized controlled trial with a 1-year follow-up showed that the PS approach appears to preserve the peri-implant bone level with a mean bone loss of 0.68 ± 0.88 mm. The PM concept had a significantly higher bone loss than the non-conventional design (2.23 ± 0.22 mm) [10].

A study of 15 articles concluded that the PS technique is essential to diminish crestal bone loss around dental implants and stressed a need for more randomized controlled clinical trials with bigger samples. However, abutment mismatches >0.45 mm demonstrated better performance [11]. Another 1-year prospective study of 26 PS bone-level dental implants and 26 PM tissue-level dental implants concluded that both approaches presented homogeneous bone loss in the lower jaw, specifically in the anterior aspect of the dental arch [12]. A recent prospective randomized clinical study reported that PS and PM dental implants executed equal radiographic and clinical performance after 1-year functional loading [13].

A prospective split-mouth study showed that the peri-implant bone loss around the PS dental implant restored at the sub-crestal level was 0.3 ± 0.2 mm after 3-year post-loading and emphasized the necessity for a long-term clinical study. The same authors noted that bleeding on probing and probing depth parameters were equally stable around dental implants [14]. A 2-year clinical and radiographic assessment examined peri-implant marginal bone loss using digital periapical radiographs, and the results suggested that subcrestally placed PS dental implants appear to be beneficial in bone with bone loss reduction around dental implants [15].

The 5-year results of a randomized clinical trial demonstrated similar interproximal bone reduction, yet minimal around PM and PS dental implants, with bone losses of 0.41 ± 0.47 mm and 0.38 ± 0.61 mm, *p* = 0.201, respectively [16]. A recent 60-month study evaluated two-piece PS dental implants that were placed in the posterior area and found marginal bone changes of 0.14 ± 0.34 mm at the mesial site and 0.26 ± 0.47 mm at the distal side, and the crown outline was not linked to marginal bone loss (MBL) and soft tissue health [17].

Various studies have shown the distinction of PS dental implants in maintaining peri-implant bone compared with PM dental implants. However, long-term studies with varying parameters, such as opposing structures of the dental implant and radiographic bone-to-implant interface contact, have not been conducted; thus, further research is needed. Hence, the 5-year effect of the PS and PM concepts on vertical and horizontal marginal bone loss is still unclear. To date, no studies have examined the effect of opposing structures on marginal bone loss around PS and PM dental implants over 5 years. Thus, this study aimed to evaluate both vertical and horizontal bone defects by using digital periapical radiographs to examine MBL changes around PM and PS dental implants over 5 years after functional loading. The null hypothesis was as follows: There was no difference in the vertical and horizontal marginal bone losses in participants who received PM or PS dental implants, against the alternative hypothesis of a difference.

## 2. Materials and Methods

**Figure 1:**
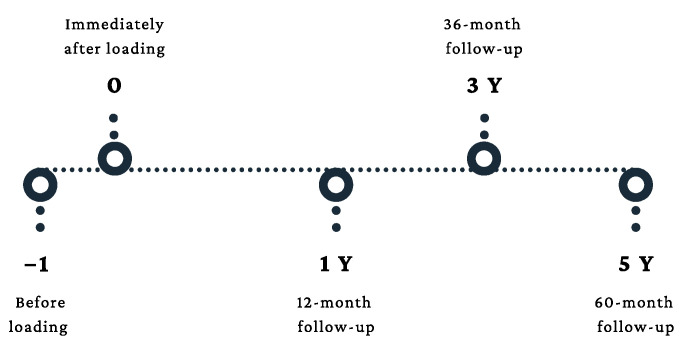
Timeline diagram.

In this retrospective cohort study, the study cohort was obtained from patients who had at least one dental implant at the Shuang-Ho Hospital, Taipei Medical University, Taiwan. The properties of this cohort have been previously published [18] and therefore will be elucidated briefly here. The study protocol was authorized by the Joint Institutional Review Board of Taipei Medical University (approval no. N202103105). Opposing structures were arranged into two groups: natural tooth and dental prostheses, which included the implant tooth and fixed and removable partial prostheses. Moreover, all implant-related parameters were documented before loading (baseline), immediately after loading (after loading = prosthetic delivery), and 1 year, 3 years, and 5 years after prosthetic loading (Figure 1).

### 2.1. Inclusion and Exclusion Criteria

We followed the instituted criteria of the American Academy of Periodontology, and adults aged >18 years who were treated with a dental implant were included in the study [19]. Patients with different opposing structures, such as natural teeth (NT) and dental prosthesis (removable and fixed restorations) against the dental arch, were included. No systemic conditions, non-smokers or smokes <10 cigarettes per day, no parafunctional habits, good oral health, no inflammation surrounding the operation site, sufficient bone tissue, and keratinized tissue level >2 mm at the time of implant surgery [20] were additional inclusion criteria.

The exclusion criteria were as follows: post and current IV therapy with amino-bisphosphonates; radiotherapy in the head area <12 months before the study; betel nut and tobacco use; smoking >10 cigarettes a day; alcohol dependence; breastfeeding and pregnancy; periodontal disease, bruxism, and clenching; excessive bone augmentation before implant surgery; persistent oral medication; reluctance to come again for follow-up oral radiographs.

### 2.2. Surgical Procedure

In this study, two kinds of titanium dental implants (Implantium, Dentium, USA, for the PS design (PS group); Xive, Dentsply, Mannheim, Germany, for the PM design (PM group)) were used and placed based on the manufacturer’s specifications and the recommended standards when there is no modification in the surrounding crestal bone tissue. Healing abutments were placed, and impressions were taken in after the insertion of the dental implants with the healing screw.

Overall, 19 individuals received 30 PM dental implants (diameter range, 3.3–4.8 mm; length range, 10–12 mm); conversely, 19 individuals received 31 PS dental implants (diameter range, 3.4–4.5 mm with an implant–abutment mismatch of 0.3 and 0.6 mm; length range, 9.5–13 mm).

Briefly, implant insertion was followed by implant dentistry control of post-surgical MBL, and a sub-crestal was placed within 1 mm from the outermost margin of the dental implants [19]. An experienced oral surgeon performed the procedure and the prosthodontic restoration process. The permanent cement-retained porcelain fused-to-metal restorations (crowns) were delivered 2 weeks after the impression and followed up for 5 years after delivering the prostheses.

### 2.3. Measurements of MBL

Outcome measurements for each radiograph were taken at the mesial and distal aspects of the dental implants. Digital periapical radiographs with film holders were used for dental implant images, and data were recollected for routine assessment. The periapical lone cone parallel technique was used for the performance of the standardized radiographs. The professional EZ dental imaging program from Asahi Roentgen IND. Co. Ltd., Kyotoy, Japan was utilized to calculate the measurement at mesial and distal sites, and the calibration part was used to rectify any digression of the periapical film.

**Figure 2:**
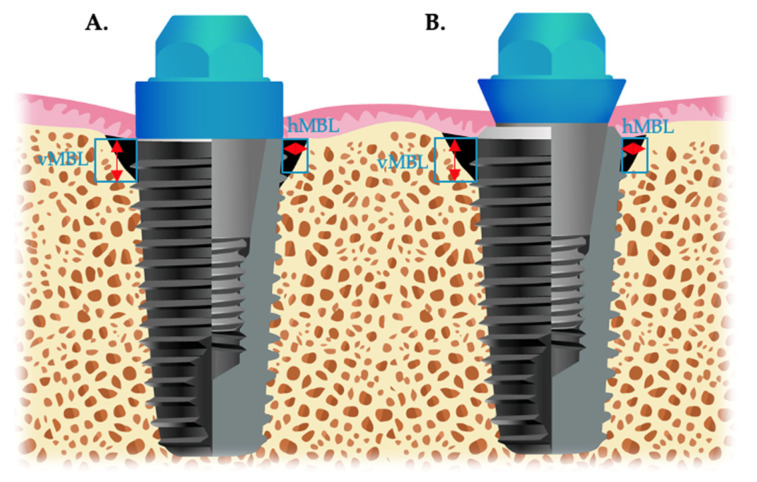
Radiographic measurements of the platform-switched and platform-matched dental implants. (**A**) Platform-matched dental implants (PM). (**B**) Platform-switched dental implants (PS). vMBL, vertical marginal bone level; hMBL, horizontal marginal bone level.

Two calibrated professionals assembled the data, and an oral surgeon performed the implant surgery. The width and length of the dental implants were used for periapical film calibration. Then, the measuring tool obtained the vertical and horizontal MBL (vMBL and hMBL, respectively). The average mesiodistal MBL were obtained for each dental implant. Linear measurements in the radiographs were taken and recorded before loading, immediately after loading (after loading), 1, 3, and 5 years (Figure 2).

The vMBL was measured from the implant–abutment to the first bone-to-implant contact [21], and hMBL was measured from the implant–abutment to the bone crest [22]. The ratio of the bone-to-implant interface contact was calculated from the vertical marginal bone change and the actual length of the dental implant [23].

Changes in vMBL and hMBL were measured by comparing the mean MBL before restoration with the MBL at different follow-up periods. The average change in the mesiodistal bone levels was calculated for each dental implant. A positive MBL suggested a loss in MBL over 5 years. A negative MBL implied gains in MBL over time.

### 2.4. Statistical Analysis

GraphPad Prism software version 8.0 for Mac (GraphPad Software, San Diego, CA, USA) was used for all data analyses. All data were presented as means ±standard errors. Paired and independent *t*-tests with Welch’s correction were used to analyze data, and comparisons were computed among and between groups for variables. For all statistical tests, the significance level was set at *p* < 0.05.

## 3. Results

This study was composed of 38 participants with 61 dental implants, which were grouped into 31 PS dental implants and 30 PM dental implants. Overall, the mean change in vMBL was 0.45 mm for the PM group and −0.43 mm for the PS group on a follow-up of 60 months. Meanwhile, the mean change in horizontal bone levels (hMBL) was 0.19 mm for the PM group and −0.40 mm for the PS group. The demographic data for the study variables and implant distribution according to study groups are demonstrated in Table 1.

### 3.1. Marginal Bone Level

In total, 19 participants had 30 PS dental implants, whereas the other 19 had 31 PM dental implants. The mean vMBL in the PM group was 1.33 ± 0.14 mm after 5-year loading, whereas it was 1.28 ± 0.21 mm in the PS group (Table 2), and the difference was not significant among the groups.

An increase in vertical marginal bone loss was observed in the PM group, i.e., from 0.88 ± 0.17 mm at baseline to 0.93 ± 0.11 at 1 year (*p* = 0.793) and to 1.03 ± 0.13 mm at 3 years (*p* = 0.485).

On the contrary, a PS dental implant was significantly more likely to lose marginal bone than PM dental implants. A decrease in vertical marginal bone loss was observed in the PS group, i.e., from 1.67 ± 0.24 mm at baseline to 1.10 ± 0.18 mm at 1 year (*p* = 0.016) and to 1.10 ± 0.18 mm at 3 years (*p* = 0.014).

However, a higher vertical marginal bone loss was observed with PS dental implants (1.67 ± 0.24 mm) than with PM dental implants (0.88 ± 0.17 mm) in the early healing period.

In addition, a similar trend was observed in the horizontal marginal bone loss around PS and PM dental implants. The mean hMBL in the PM group was 1.04 ± 0.08 mm after 5-year loading, whereas it was 0.76 ± 0.10 mm in the PS group (Table 3).

A slight increase in horizontal marginal bone loss was observed in the PM group, i.e., from 0.84 ± 0.13 mm at baseline to 0.93 ± 0.11 at 3 years (*p* = 0.606).

By contrast, a PS dental implant was significantly more likely to lose bone than PM dental implants. A decrease in horizontal marginal bone loss was observed in the PS group, i.e., from 1.20 ± 0.19 mm at baseline to 0.67 ± 0.08 mm at 3 years, which was significant (*p* = 0.011). Moreover, the horizontal marginal bone loss was significant after 5-year loading among the PM group (*p* = 0.021).

However, a higher horizontal marginal bone loss was observed with PS dental implants (1.20 ± 0.19 mm) than with PM dental implants (0.84 ± 0.13) in the early healing period.

**Figure 3:**
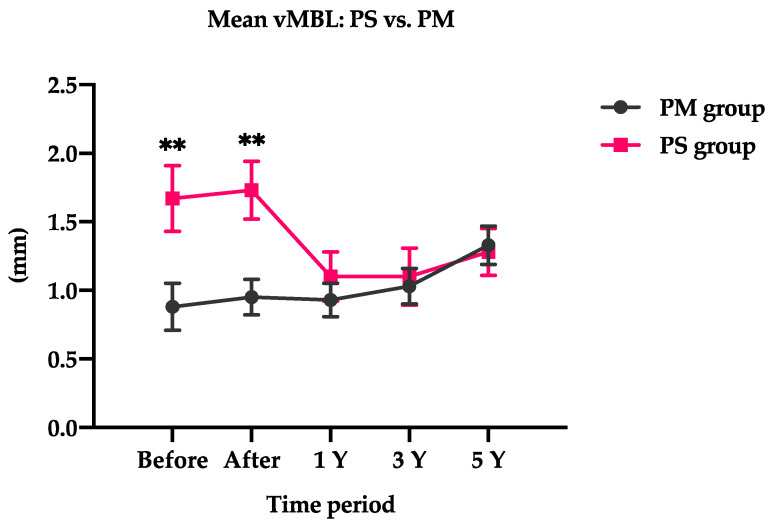
Comparison of mean vMBL between the PS and PM groups. PS, platform-switched dental implants; PM, platform-matched dental implants; vMBL, vertical marginal bone level. ** *p* < 0.01.

A higher marginal bone loss was noted in the PS group in the early healing period than in the PM group, which was significant before loading (*p* = 0.009) and immediately after loading (*p* = 0.003) as shown in the Figure 3. Over time, PS dental implants are more likely to gain bone; by contrast, PM dental implants are more likely to lose bone during the observation time.

**Figure 4:**
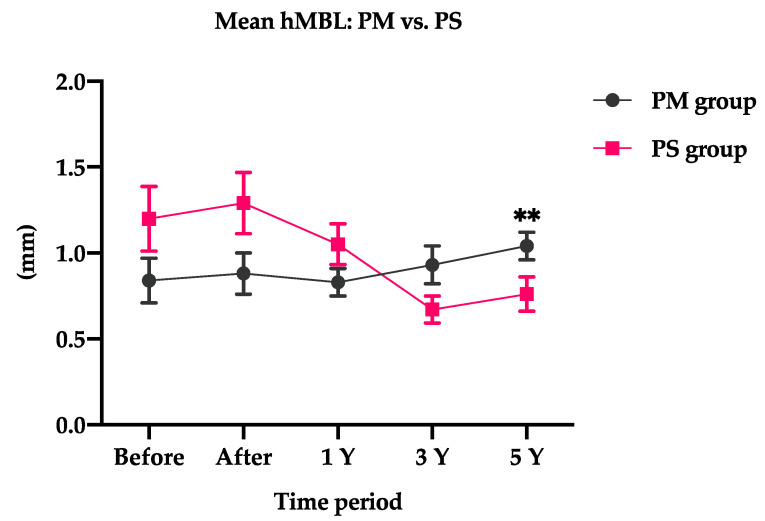
Comparison of the mean hMBL between the PS and PM groups. PS, platform-switched dental implants; PM, platform-matched dental implants; hMBL, horizontal marginal bone level. ** *p* < 0.01.

Interestingly, a similar MBL trend was observed in the PM and PS groups. The MBL in the PM group increased from 1 year after loading, whereas the MBL in the PS group decreased immediately after loading (Figure 4). Over time, PS dental implants were more likely to gain bone, whereas PM dental implants were more likely to lose bone during the observation time, and the MBL after 5-year loading was significant between the PM and PS groups, with 1.04 ± 0.43 mm and 0.76 ± 0.54 mm (*p* = 0.036), respectively.

### 3.2. Change in Marginal Bone Level

Almost similar changes were observed in the vMBL in the PS and PM groups immediately after loading, which implied bone loss in both groups. In PM dental implants, vMBL changes were increasing over time; however, the changes in PS dental implants were declining over the years (Table 4).

At 1-year loading, the changes in vMBL for PM and PS dental implants were 0.06 ± 0.21 mm and −0.57 ± 0.22 mm, respectively. PS dental implants were significantly less likely to lose bone at 1-year loading (*p* = 0.045). Similarly, at 3-year loading, changes in vMBL for PM and PS dental implants were 0.16 ± 0.22 mm and −0.61 ± 0.23 mm, respectively. PS dental implants were significantly less likely to lose bone at 3-year loading (*p* = 0.021). Moreover, at 3-year loading, changes in vMBL for PM and PS dental implants were 0.45 ± 0.23 mm and −0.43 ± 0.24 mm, respectively, which was also significant between the groups (*p* = 0.021).

At 1-year loading, the changes in the hMBL for the PM and PS dental implants were −0.01 ± 0.12 mm and −0.15 ± 0.17 mm, respectively. Over time, PS dental implants were significantly less likely to lose bone (Table 4). The changes in the hMBL for PM and PS groups after 3-year loading were 0.08 ± 0.16 mm and −0.49 ± 0.18, respectively (*p* = 0.021). The changes in the hMBL for the PM and PS groups after 5-year loading were 0.19 ± 0.13 mm and −0.40 ± 0.16, respectively, which was significant (*p* = 0.006).

Furthermore, the change in the mesial vMBL had almost similar amounts of bone immediately after loading for the PS and PM groups. Over time, the mesial vMBL in the PM group was losing more bone, whereas the mesial vMBL in the PS group was gaining bone. The group analysis revealed that mesial vMBL were significant at 1-year (*p* = 0.008), 3-year (*p* = 0.002), and 5-year (*p* = 0.037) loading. However, the mesial change in the PM group did not reveal any significance over time (Table 5).

Moreover, the change in the hMBL had a nearly similar trend as vMBL. The change in hMBL was significantly different among groups at 3-year (*p* = 0.002) and 5-year (*p* = 0.004) loading in the PS group (Table 5).

A higher average bone-to-implant interface contact was observed in the PM group at baseline (92.1 ± 1.56%) than in the PS group, which was decreasing over time, insignificantly (Table 6). An average bone-to-implant interface contact was noted in the PS group at baseline (83.7 ± 2.30%), which was modified significantly over time immediately after loading (*p* < 0.001) and at 1-year (*p* < 0.001), 3-year (*p* < 0.001), and 5-year (*p* < 0.001) loading.

### 3.3. Opposing Structures

**Figure 5:**
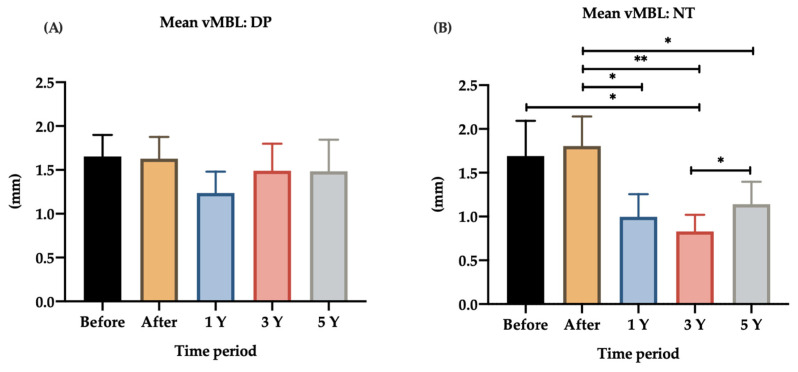
Bar chart of the mean vMBL around PS dental implants opposing DP and NT. vMBL, vertical marginal bone level; DP, dental prostheses; NT, natural teeth; 1 Y, 1-year follow-up; 3 Y, 3-year follow-up; 5 Y, 5-year follow-up. * *p* < 0.05; ** *p* < 0.01. (**A**) Mean vMBL in the DP group; (**B**) mean vMBL in the NT group (before vs. 3-year loading, *p* = 0.023; after vs. 1-year loading, *p* = 0.011; after vs. 3-year loading, *p* = 0.003; after vs. 5-year loading, *p* = 0.029; 3-year vs. 5-year loading, *p* = 0.023).

**Figure 6:**
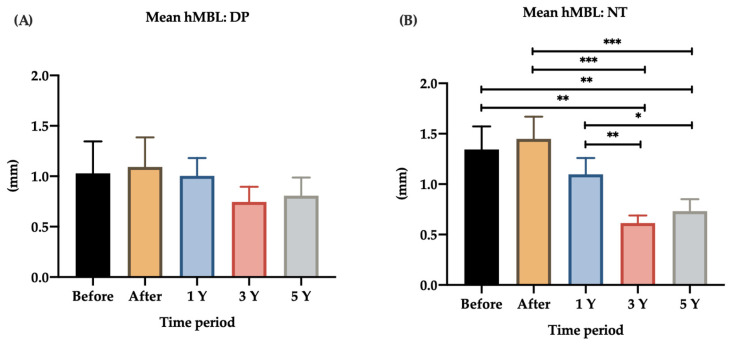
Bar chart of the mean hMBL around PS dental implants opposing DP and NT. hMBL, horizontal marginal bone level; DP, dental prostheses; NT, natural teeth; 1y, 1-year follow-up; 3y, 3-year follow-up; 5y, 5-year follow-up. * *p* < 0.05; ** *p* < 0.01; *** *p* < 0.001. (**A**) Mean hMBL in the DP group; (**B**) Mean hMBL in the NT group (before vs. 3-year loading, *p* = 0.002; before vs. 5-year loading, *p* = 0.002; after vs. 3-year loading, *p* < 0.001; after vs. 5-year loading, *p* < 0.001; 1-year vs. 3-year loading, *p* = 0.006; 1-year vs. 5-year loading, *p* = 0.023).

In this analysis, only PS dental implants opposed by NT and dental prosthesis were included. In total, 17 dental implants were opposed by NT and 14 dental implants by dental prostheses. Dental prostheses include implant-supported restoration, fixed restorations, and removable partial prosthesis. Significant differences were found in the MBL over time (vMBL and hMBL) of dental implants that were opposed by NT (Figure 5 and Figure 6).

For dental implants opposing NT, the average vMBL was 1.69 ± 0.40 mm at baseline and 1.81 ± 0.34 mm immediately after loading. PS dental implants were significantly more likely to lose bone if they were opposed by NT (before vs. 3-year loading, *p* = 0.023), whereas the PS dental implants opposed by DP did not reach significance (before vs. 3-year loading, *p* = 0.376). Furthermore, the vMBL immediately after loading was modified significantly, as follows: after vs. 1-year loading, *p* = 0.011; after vs. 3-year, *p* = 0.003; after vs. 5-year loading, *p* = 0.029; 3-year vs. 5-year loading, *p* = 0.023.

For dental implants opposing NT, the average hMBL was 1.34 ± 0.23 mm at baseline and 1.45 ± 0.22 mm immediately after loading. PS dental implants were significantly more likely to lose bone if they were opposed by NT (before vs. 3-year loading, *p* = 0.002; before vs. 5-year loading, *p* = 0.002), whereas the PS dental implants opposed by DP did not reach significance (before vs. 3-year loading, *p* = 0.641; before vs. 5-year loading, *p* = 0.762). Furthermore, the vMBL immediately after loading was modified significantly as follows: after vs. 3-year loading, *p* < 0.001; after vs. 5-year loading, *p* < 0.001; 1-year vs. 3-year loading, *p* = 0.006; 1-year vs. 5-year loading, *p* = 0.023.

## 4. Discussion

Throughout the 5-year study period, the PS concept had lower marginal bone loss than the PM concept. PS dental implants revealed greater resorption based on the mean vertical bone loss at the end of the 1-year observation (1.10 ± 0.18 mm) than PM dental implants (0.93 ± 0.12 mm). Consistent outcomes have been demonstrated in previous studies, with a lesser crestal bone loss in PM dental implants and a greater bone loss in PS dental implants.

Rashmita Nayak et al. evaluated the crestal bone loss around different platform dental implants, which corresponded to the present study. A 12-month retrospective study found that the mean vertical crestal bone level for PS dental implants was 2.16 ± 1.02 mm (range, 2.16–3.12 mm), whereas the average for PM dental implants was 1.55 ± 0.82 mm (range, 1.55–4.18 mm) of crestal bone loss. In addition, the mean horizontal crestal bone level for PS dental implants was 2.27 ± 0.91 mm, whereas the mean crestal bone level was 2.16 ± 0.77 mm for the PM dental implants [24].

Moreover, the results of a randomized controlled trial found significantly lower and better crestal bone levels in the PS groups [25]. Cappiello et al. also reported a vertical bone gap of 0.95 ± 0.32 mm (range, 0.6 mm–1.2 mm) for the PS dental implants. For the PM dental implants, the bone gap was 1.67 ± 0.37 mm (range, 1.3–2.1 mm). Thus, a minimal bone gap was noted in PS dental implants, whereas a mean bone gap of 1 mm to 2 mm was found in PM dental implants [26].

The tendency for vertical and horizontal marginal bone losses for the PS dental implants reduced over time, which was consistent with the results of a previous study [24]. However, with extended observation time, both PS and PM dental implants exhibited matching results in the present study. On the contrary, the vertical and horizontal bone loss for the PM dental implants increased over time; this may have been be due to the varying types of dental implants used and the longer follow-up time. Moreover, the PS concept helps maintain long-term esthetic outcomes by reducing the vMBL and hMBL [27,28].

A 5-year randomized controlled trial with a total of 202 dental implants indicated that the mean MBL for PM dental implants was 0.26 ± 0.55 mm at 1 year and 0.61 ± 0.73 mm at 5 years. Meanwhile, the mean MBL for PS dental implants was −0.03 ± 0.74 mm at 1 year and −0.20 ± 0.75 mm at 5 years [29].

MBL changes of 0.06 ± 0.21 mm and 0.45 ± 0.23 mm were noted after the 1-year and 5-year follow-up, respectively, for the control group. In the test group, MBL change was −0.57 ± 0.22 mm at 1-year and −0.43 ± 0.24 mm at 5-year observation, which was even lower than that in the previous study. Similar to those reported by Guerra et al. [30] after 1-year loading, with 0.08 ± 0.41 mm of gain for the test group and −0.06 ± 0.81 mm of bone loss for the control group, Ana et al. reported 0.19 ± 0.53 mm gain for the test group and −0.04 ± 0.58 mm of bone loss for the control group after 5-year loading [31].

Furthermore, in the present study, the 3-year MBL results (test group, 1.10 ± 0.18; control group, 1.03± 0.13) corresponded to those noted by Pan et al. (test group, 0.78 ± 0.77 mm; control group, 0.98 ± 0.81 mm) [18] and were inconsistent with those of Rocha et al. (test group, 0.28 ± 0.56 mm; control group, 0.68 ± 0.64 mm) [32].

A systematic review of 26 studies indicated that PS dental implants were more likely to have protective effects on hard tissue around dental implants than PM dental implants [33].

Another systematic review with a meta-analysis of 15 studies concluded that the PS method helped reduce crestal bone loss around dental implants, and dental implants with mismatches >0.45 mm revealed better results. The authors suggested that more clinical trials and greater sample sizes are necessary to confirm the outcome [11].

In a recent systematic review and meta-analysis of nine studies with 1-year observation period, PS dental implants demonstrated lesser crestal bone loss than non-PS dental implants [34]. In a randomized clinical trial with 5-year follow-up, trends of MBL decreasing and increasing were observed for PS and PM dental implants, respectively [29]. Moreover, most of the studies comparing PS and PM dental implants reported that dental implants with switching platforms exhibited a lesser marginal bone reduction.

A prospective study compared the peri-implant bone loss around PS and PM dental implants. The authors found 0.22 mm of mean bone loss for the PS group and 2.02 mm for the non-PS group [35]. Fickl et al. examined 98 dental implants and found marginal bone remodeling after 1 year of follow-up, and the bone levels were 0.39 and 1.00 mm for the PS and PM methods, respectively [36].

These findings were inconsistent with those of the present study, as an increasing trend was noted in both vMBL and hMBL, whereas a reduction was observed in both groups.

After the 5-year follow-up, the present study revealed an MBL change of 0.45 ± 0.23 mm for the control group, similar to those demonstrated by Lago et al. (0.61 ± 0.73 mm), Kielbassa et al. (0.63 ± 1.18 mm), and Enkling et al. (0.61 ± 0.57 mm) [29,37,38]. In addition, the MBL change for the test group in the present trial was −0.43 ± 0.24 mm over 5 years, similar to the outcomes reported by Lago et al. (−0.28 ± 0.45 mm), Messias et al. (0.19 ± 0.53 mm), Canullo et al. (0.37 ± 0.12 mm), Kielbassa et al. (0.30 ± 0.16 mm), Pieri et al. (0.20 ± 0.17 mm), Herekar et al. (−0.34 mm), and Hürzeler et al. (−0.22 ± 0.53 mm) [25,31,35,37,39,40,41].

Although many studies have evaluated PS and PM dental implants, the follow-up did not transcend 3 years of observation [32,42,43,44]. A few studies have reported 5-year data; only one study compared PM and PS dental implants, and the 5-year outcomes of the MBL change were similar to those in the present study. The results from other 5-year studies were not definitive due to the absence of a PM concept, and none of them analyzed the contributing factors of bone modification, such as the opposing structure of dental implants [31,39,45,46].

Both dental implant systems had similar results, with bone loss for the PM design and bone gain for the PS design noted over the 5-year follow-up period. However, the experimental group appeared to exhibit greater marginal bone preservation over time, with some alterations. In addition, significant modifications were noted in the vMBL from 1-year post-loading, whereas significant changes were exhibited in hMBL. In addition, a higher bone-to-implant interface contact was noted in PS dental implants than in the non-PS dental implants over 5 years. Moreover, significant differences were observed in the MBL of PS dental implants if they were opposed by natural teeth and if they were opposed by dental prostheses. The marginal bone level difference was not noted between these two opposing conditions in the present study. These opposing conditions results were in agreement with the previously published study. Authors found 0.30 mm of MBL for the natural teeth group and 0.53 mm of MBL for the fixed restoration group after 36-month loading [47]. Although the level of bone loss was slightly higher in the present study, lower marginal bone loss noted in the natural teeth group when compared with the dental prosthesis.

This study is mainly limited by its retrospective study design, small sample population, use of two-dimensional (2D) digital radiographs, and a single center for implant insertion. Complete data are necessary for retrospective cohort study, and because of possible confounders and selection bias; thus, the study design has less validity than other study designs, such as randomized prospective clinical trials. Another drawback is the use of 2D radiographs only for linear measurements; thus, volumetric measurement of the crestal bone cannot be employed around dental implants. In the literature, marginal bone change around the apex of the dental implants can be observed [48]. The power of the study would be increased by larger sample sizes; thus, multiple centers, randomized, prospective clinical trials are necessary to validate the present outcomes.

## 5. Conclusions

Within the aforementioned limitations, the PS concept appears to preserve more MBL around dental implants by stabilizing the mean vMBL and hMBL over 5 years. Moreover, minimal marginal bone change was detected in both PS and non-PS dental implants.

## Figures and Tables

**Table 1:** Demographic parameters of the study participants.

Parameter	PS Group (*n* = 31)	PM Group (*n* = 30)
Sex, *n* (%)		
Female	19 (61.3)	14 (46.7)
Male	12 (38.7)	16 (53.3)
Implant diameter *n* (%)		
3.3 mm	4 (12.9)	0
3.4 mm	0	3 (10.0)
3.8 mm	12 (38.7)	20 (66.7)
4.3 mm	12 (38.7)	0
4.5 mm	0	7 (23.3)
4.8 mm	3 (9.7)	0
Implant length *n* (%)		
9.5 mm	0	5 (16.7)
10 mm	28 (90.3)	0
11 mm	0	17 (56.7)
12 mm	3 (9.7)	0
13 mm	0	8 (26.7)
Opposing structure *n* (%)		
NT	17 (54.8)	8 (26.6)
DP	14 (45.2)	22 (73.3)

Note: PS, platform-switched; PM, platform-matched; NT, natural teeth; DP, dental prostheses.

**Table 2:** The mean vMBL outcomes.

Time	PM Group	*p* Value	PS Group	*p* Value
	Mean ± SEM		Mean ± SEM	
Before	0.88 ± 0.17		1.67 ± 0.24	
After	0.95 ± 0.13	0.686	1.73 ± 0.21	0.533
1 year	0.93 ± 0.11	0.793	1.10 ± 0.18	0.016 *
3 years	1.03 ± 0.13	0.485	1.10 ± 0.18	0.014 *
5 years	1.33 ± 0.14	0.057	1.28 ± 0.21	0.084

Note: vMBL, vertical marginal bone level; PS, platform-switched dental implants; PM, platform-matched dental implants; SEM, standard error of mean. * *p* < 0.05.

**Table 3:** Mean hMBL outcomes of the PM and PS groups.

Time	PM Group	*p* Value	PS Group	*p* Value
	Mean ± SEM		Mean ± SEM	
Before	0.84 ± 0.13		1.20 ± 0.19	
After	0.88 ± 0.11	0.658	1.29 ± 0.18	0.409
1 year	0.83 ± 0.08	0.914	1.05 ± 0.12	0.401
3 years	0.93 ± 0.11	0.606	0.67 ± 0.08	0.011 *
5 years	1.04 ± 0.08	0.149	0.76 ± 0.10	0.021 *

Note: hMBL, horizontal marginal bone level; PM, platform-matched dental implants; PS, platform-switched dental implants; SEM, standard error of mean; * *p* < 0.05.

**Table 4:** Comparison of the mean vMBL and hMBL change between the two groups.

The Mean vMBL Change			
Time	PM Group	PS Group	*p* Value
	Mean ± SEM	Mean ± SEM	
After	0.07 ± 0.18	0.05 ± 0.08	0.918
1 year	0.06 ± 0.21	−0.57 ± 0.22	0.045 *
3 years	0.16 ± 0.22	−0.61 ± 0.23	0.021 *
5 years	0.45 ± 0.23	−0.43 ± 0.24	0.010 *
**The Mean hMBL Change**			
**Time**	**PM Group**	**PS Group**	***p* Value**
	**Mean ± SEM**	**Mean ± SEM**	
After	0.04 ± 0.09	0.09 ± 0.10	0.739
1 year	−0.01 ± 0.12	−0.15 ± 0.17	0.531
3 years	0.08 ± 0.16	−0.49 ± 0.18	0.021 *
5 years	0.19 ± 0.13	−0.40 ± 0.16	0.006 **

Note: vMBL, vertical marginal bone level; PS, platform-switched dental implants; PM, platform-matched dental implants; SEM, standard error of mean; * *p* < 0.05; ** *p* < 0.01.

**Table 5:** The vMBL and hMBL change at the mesial site of the PM and PS groups.

The vMBL Change at the Mesial Site				
Time	PM Group	*p* Value	PS Group	*p* Value
	Mean ± SEM		Mean ± SEM	
After	0.10 ± 0.18		0.11 ± 0.08	
1 year	0.11 ± 0.24	0.913	−0.54 ± 0.25	0.008 **
3 years	0.22 ± 0.22	0.379	−0.62 ± 0.24	0.002 **
5 years	0.46 ± 0.26	0.552	−0.39 ± 0.26	0.037 *
**The hMBL Change at the Mesial Site**				
**Time**	**PM Group**	***p* Value**	**PS Group**	***p* Value**
	**Mean ± SEM**		**Mean ± SEM**	
After	0.15 ± 0.12		−0.06 ± 0.14	
1 year	−0.01 ± 0.21	0.436	−0.32 ± 0.25	0.366
3 years	0.03 ± 0.19	0.731	−0.83 ± 0.25	0.002 **
5 years	0.19 ± 0.16	0.808	−0.72 ± 0.24	0.004 **

Note: vMBL, vertical marginal bone level; PS, platform-switched dental implants; PM, platform-matched dental implants; SEM, standard error of mean. * *p* < 0.05; ** *p* < 0.01.

**Table 6:** Mean r-BIIC percentage of the PM and PS groups.

Time	PM Group	*p* Value	PS Group	*p* Value
	Mean ± SEM		Mean ± SEM	
Before	92.1 ± 1.56		83.7 ± 2.30	
After	93.3 ± 1.07	0.418	96.7 ± 0.55	<0.001 ***
1 year	92.9 ± 1.36	0.557	90.3 ± 1.65	<0.001 ***
3 years	91.1 ± 1.17	0.445	90.9 ± 1.85	<0.001 ***
5 years	89.6 ± 1.23	0.176	91.5 ± 1.86	<0.001 ***

Note: r-BIIC%, radiographic bone-to-implant interface contact; PS, platform-switched dental implants; PM, platform-matched dental implants; SEM, standard error of mean. *** *p* < 0.001.

## Data Availability

Not applicable.
